# The effective doses of remimazolam besylate in the procedural sedation of endoscopic retrograde cholangiopancreatography

**DOI:** 10.1002/ibra.12072

**Published:** 2022-11-09

**Authors:** Dan‐Dan Tan, Jin Gu, Juan Li, Wan‐Qiu Yu, De‐Xing Liu, Li‐Jin Zhao, Guo‐Hua Zhu, Xin‐Xin Yang, Jin Tian, Qi Tian, Zhao‐Qiong Zhu

**Affiliations:** ^1^ Department of Anesthesiology Affiliated Hospital of Zunyi Medical University Zunyi Guizhou China; ^2^ Department of Hepatology Affiliated Hospital of Zunyi Medical University Zunyi Guizhou China; ^3^ Yichang Humanwell Pharmaceutical Co., Ltd Yichang Hubei China; ^4^ Department of Anesthesiology, Union Hospital, Tongji Medical College Huazhong University of Science and Technology Wuhan Hubei China; ^5^ Heidelberg University Heidelberg Germany; ^6^ Memorial Herman Hospital Houston Texas USA

**Keywords:** dose finding, ERCP, remimazolam besylate, sequential test method

## Abstract

This study aimed to determine the values of the half‐effective dose (ED_50_) and 95% effective dose (ED_95_) of remimazolam besylate used in the procedural sedation of endoscopic retrograde cholangiopancreatography (ERCP). Sixty patients who fulfilled the inclusion and exclusion criteria of this study were selected. Sufentanil was administered intravenously and remimazolam besylate was administered 2 min later. ERCP treatment was feasible when the modified alertness/sedation (MOAA/S) score was ≤2. If choking or movement occurred during duodenoscope placement, it was considered as a positive reaction. The dose was increased in the next patient; otherwise, it was considered as a negative reaction, and the dose was reduced in the next patient. The ED_50_ and ED_95_ values and 95% confidence interval (CI) of remimazolam besylate were calculated by Probit regression analysis. All 60 patients completed the trial. The ED_50_ and ED_95_ values of remimazolam besylate were 0.196 and 0.239 mg/kg, respectively, for the procedural sedation of ERCP. The time of MOAA/S score ≤ 2 was (82.58 ± 21.70) s, and the mean time of awakening was (9.03 ± 5.64) min. Transient hypotension was observed in two patients without medical intervention. The ED_50_ and ED_95_ values of remimazolam besylate used in the procedural sedation of ERCP were 0.196 and 0.239 mg/kg, and the dose of the medications has definite efficacy and good safety.

## Graphical abstract

The combination of remimazolam besylate and sufentanil in endoscopic retrograde cholangiopancreatography yields a good sedative effect and only mild adverse reactions; importantly, we calculated a half‐effective dose and 95% effective dose values for remimazolam besylate. The mechanism may be related to the action of remimazolam besylate on γ‐aminobutyric acid type‐A receptors, affecting the inward flow of chloride ions.

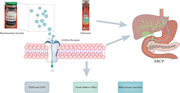

## 1 INTRODUCTION

In recent years, endoscopic retrograde cholangiopancreatography (ERCP) has become an indispensable minimally invasive intervention for the diagnosis and treatment of biliary and pancreatic diseases.^1^ However, it is complex and takes a long time for diagnosis and treatment, which leads to huge challenges in terms of surgeries.^2^ At present, ERCP is mostly performed under intravenous anesthesia, which helps maintain spontaneous breathing in clinical practice. Patients are usually placed in the prone position or semiprone position. This integration may cause hemodynamic and physiological changes in lung ventilation and may increase the difficulty of respiratory tract management during the operation.^2^, ^3^, ^4^ Therefore, we need to select an anesthetic that has the least influence on respiratory cycles. Remimazolam besylate is a new type of ultrashort‐acting benzodiazepine.^5^ It acts on the central γ‐aminobutyric acid type‐A, a receptor that opens the receptor channel and increases the internal flow of chloride ions, thus causing hyperpolarization of the nerve cell membrane to inhibit neuronal activity and produce the sedative effect. It can be rapidly hydrolyzed into an inactive carboxylic acid metabolite by tissue esterase present in the body.^6^, ^7^, ^8^ Remimazolam besylate has limited influence on respiratory inhibition, so it maintains stable hemodynamics optimally.^5^ It is mainly used for the sedation of painless gastrointestinal endoscopy. There are no reports of its effective doses for painless ERCP diagnosis and treatment in China and overseas. In this study, we aimed to carry out sequential trials to calculate the half‐effective dose (ED_50_) and the 95% effective dose (ED_95_) of remimazolam besylate in the procedural sedation of ERCP, thus evaluating its safety during anesthesia and providing a reference for rational use in clinical practice.

## 2 MATERIALS AND METHODS

### 2.1 General information

This prospective and sequentially designed study was approved by the Ethics Committee of the Affiliated Hospital of Zunyi Medical University (KLL‐2020‐280). Written informed consent was signed by the patients or their family members. The trial process was registered at chictr.org.cn (no. ChiCTR2200059783). Sixty patients who were about to receive ERCP from November 2021 to May 2022, aged 18–65 years, male or female, with a body mass index (BMI) of 18–30 kg/m^2^ and American Association of Anesthesiologists (ASA) Grade I–II were included in the study. The following patients were excluded: ① patients in whom sedatives were used within 24 h; ② patients with hypertension whose blood pressure (BP) was not satisfactorily controlled by antihypertensive drugs (systolic lying pressure ≥ 180 mmHg in the screening phase, and/or diastolic BP ≥ 110 mmHg); ③ patients with a history of severe cardiovascular and myocardial infarction in the last 6 months; ④ patients with cognitive dysfunction and mental system diseases such as depression, Alzheimer's disease, and so on; ⑤ pregnant or lactating women; ⑥ patients considered by the researchers to be unsuitable for this study.

### 2.2 Treatment procedures

Drinking and eating were prohibited before anesthesia. After entering the ERCP operating room, the patients were placed on the left side in the prone position. The venous channel was established. Oxygen was inhaled through a nasal catheter (3 L/min). BP, heart rate (HR), respiratory rate (RR), and pulse oxygen saturation (SPO_2_) were monitored and the basic data were recorded. The patients were intravenously injected with 0.1 μg/kg sufentanil (Yichang Humanwell Pharmaceutical Co., Ltd; SFDA approval number: H20054171; specification: 1 ml:50 μg) and then administered the preset dose of remimazolam besylate (Yichang Humanwell Pharmaceutical Co., Ltd; SFDA approval number: H20200006; specification: 25 mg) according to the sequential test method by an intravenous injection 2 min later. The administration time was (60 ± 10) s. When the modified alertness/sedation (MOAA/S) score was less than or equal to 2, the ERCP diagnosis and treatment could be conducted (Table 1). During the diagnosis and treatment of ERCP, a 1 mg/kg/h pump of remimazolam besylate was used for maintenance. If the diagnosis and treatment were disrupted by movements, coughing, and other factors, 0.05 mg/kg of remimazolam besylate was injected intravenously. If remimazolam besylate was supplemented more than five times within 15 min, the sedation failed, and propofol 0.5 mg/kg was used to compensate the sedation. During the diagnosis and treatment, if the systolic BP (SBP) was less than 30% of the basic value, ephedrine was used for symptomatic treatment. Atropine 0.5 mg was administered intravenously if HR ≤ 50 times/min; when SPO_2_ ≤ 93%, the patients were required to hold the lower jaw and increase the inflow of oxygen. When SPO_2_ ≤ 80%, the endoscopic operation was stopped and oxygen was further supplied through the mask. Remimazolam besylate was stopped immediately after the operation and patients were transferred to the postanesthesia care unit (PACU), where they could be returned to the ward with their family members when the Aldrete score was ≥9 (Table 2).

**Table 1:** Modified alertness/sedation (MOAA/S) scoring criteria

Grade	Standard
5 points	Fully awake and respond normally to normal first name calls
4 points	Slow to respond to normal name calls
3 points	Responding to repeated loud name calls
2 points	Reacting to slight pushing or shaking of the body
1 point	Respond to painful stimuli (squeezing of the trapezius muscle)
0 points	No response to painful stimuli (squeezing of the trapezius muscle)

**Table 2:** Aldrete scoring criteria

Evaluation project	Scoring (score)
Activity	
Move limbs as instructed	2
Move both limbs as instructed	1
Unable to move limbs as instructed	0
Breathe	
Can take deep breaths and cough freely	2
Dyspnea; respiratory distress; breathing difficulties	1
Apnea	0
Circulate	
Blood pressure fluctuation amplitude < 20% of the preanesthesia level	2
Fluctuation range of blood pressure was 20%–49% of that before anesthesia	1
Blood pressure fluctuated by 50% of the preanesthesia level	0
Consciousness	
Fully awake	2
Can wake up	1
No response	0
SPO_2_	
Breathing air > 92%	2
Need oxygen > 90%	1
<90% under oxygen inhalation	0

Abbreviation: SPO_2_, oxygen saturation.

**Figure 1:**
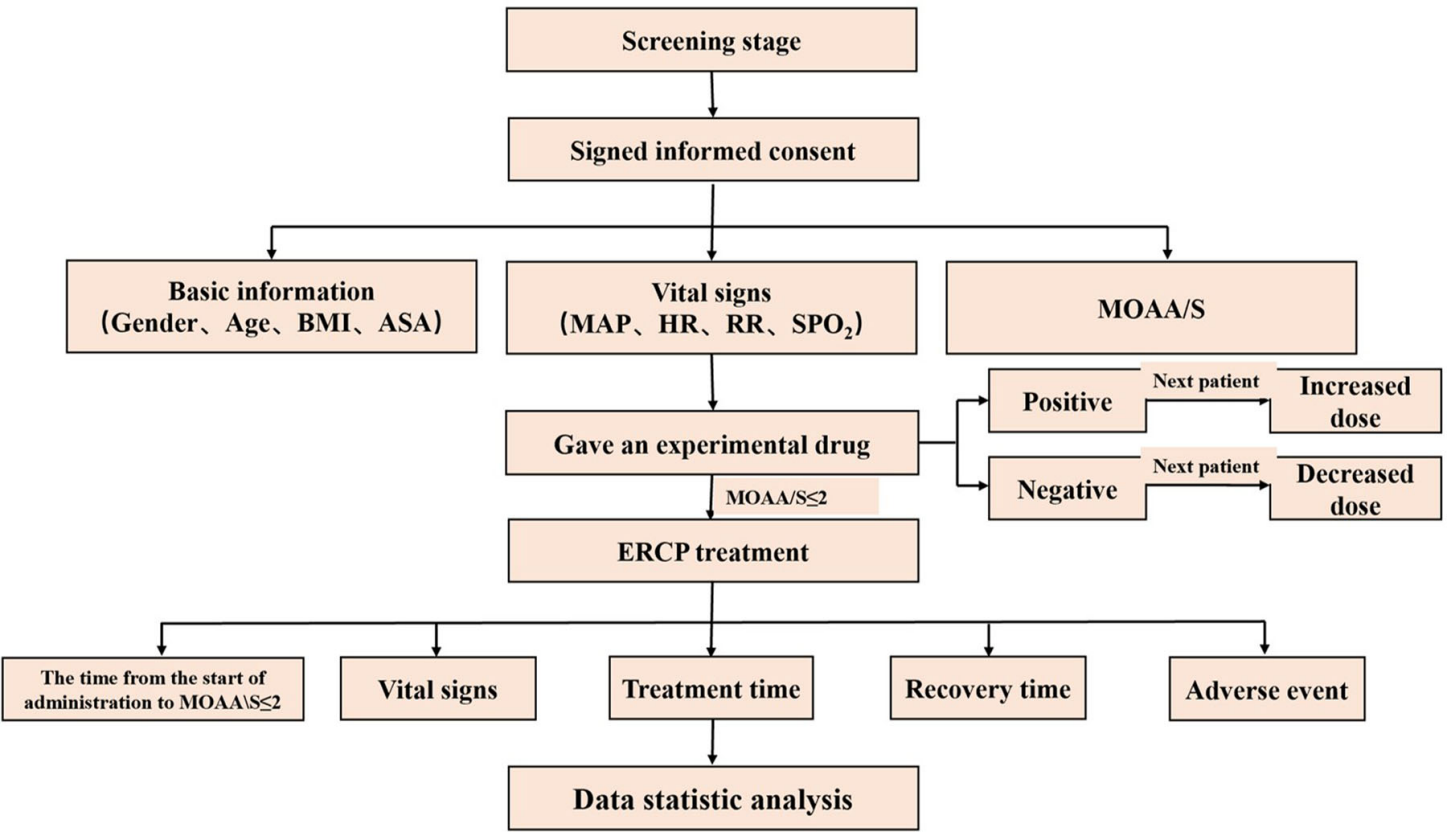
The test flowchart. A positive reaction is determined by the presence of choking and body movements during the insertion of the duodenoscope; if not, the reaction is judged to be negative. ASA, American Association of Anesthesiologists; BMI, body mass index; ERCP, endoscopic retrograde cholangiopancreatography; HR, heart rate; MAP, mean arterial pressure; MOAA/S, modified alertness/sedation; PACU, postanesthesia care unit; RR, respiratory rate; SPO_2_, pulse oxygen saturation. [Color figure can be viewed at wileyonlinelibrary.com]

According to the sequential test method, based on the pretest and previous studies,^9^ the dose of the first patient was 0.15 mg/kg. The drug dose for the next patient was determined by the reaction of the previous patient. If cough and body movement occurred during the placement of the duodenoscope, they were determined to be positive reactions, and the dose for the next patient was increased. On the contrary, if no cough and body movement occurred during the placement of the duodenoscope, they were considered to be negative reactions, and the dose for the next patient was decreased. The dose gradient was 0.02 mg/kg. The test flowchart is presented in Figure 1.

### 2.3 Observation of relevant indicators

*Main indicators*: The effective sedation dose of remimazolam besylate was determined. Recorded the time from the initiation of remimazolam besylate to MOAA/S score ≤ 2. Recorded the awakening time of the patient (MOAA/S score ≥ 4).

Secondary indicators included perioperative adverse events. The vital signs (BP, HR, RR, and SPO_2_) of the patients were observed and recorded: the basic value after they entered the room (*T*_0_), 2 min after starting the administration of remimazolam besylate (*T*_1_), when the duodenoscopy was performed (*T*_2_), 5 min after the duodenoscopy was performed (*T*_3_), at the end of diagnosis and treatment (*T*_4_), when the patient woke up (*T*_5_), and when the patient left the PACU (*T*_6_). Adverse reactions such as injection pain, hypotension, nausea, and vomiting after drug administration were recorded.

### 2.4 Statistical analysis

SPSS 18.0 statistical software was used for statistical analysis. Data of normal distributions were expressed as mean ± standard deviation (SD). Enumeration data were expressed as frequency, and the *t*‐test was used for comparison between groups. *p* < 0.05 indicated a significant difference. ED_50_ and ED_95_ values, and the 95% confidence interval (95% CI) were calculated using Probit regression analysis $\bar{x}\pm \mathrm{SD}$.

## 3 RESULTS

### 3.1 ED_50_ and ED_95_ values of remimazolam besylate used in the procedural sedation of ERCP

**Figure 2:**
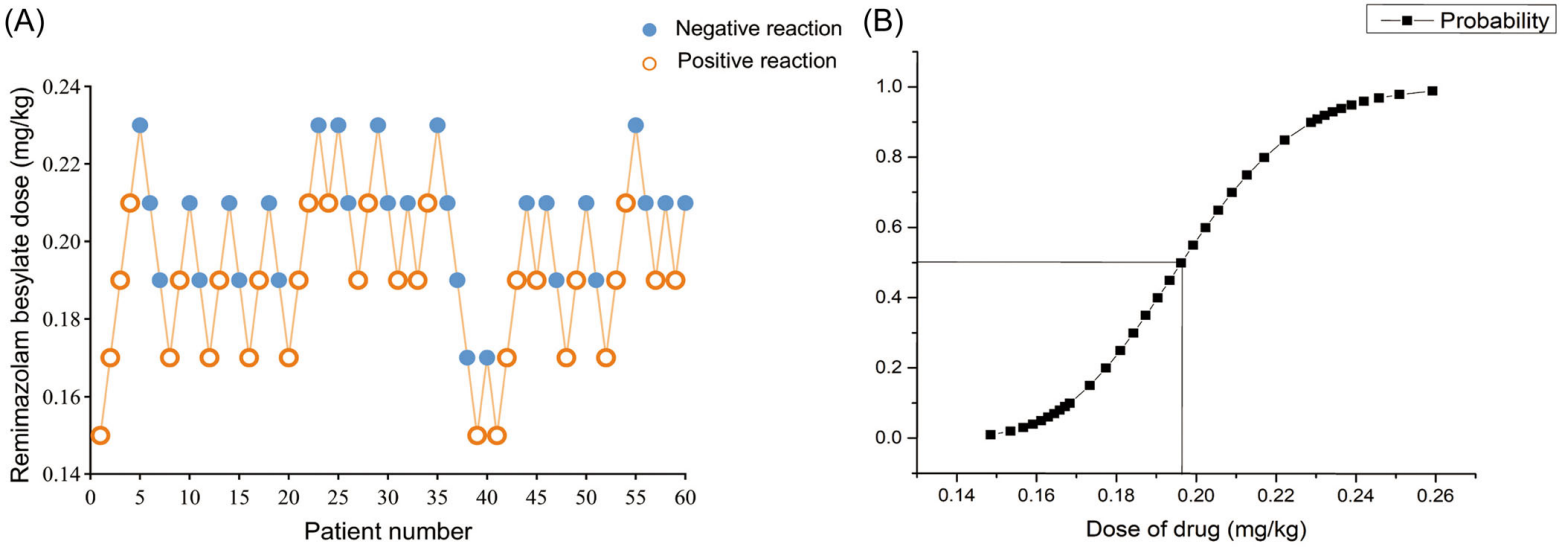
Sequential experimental diagram and dose–effect curve fitting diagram of remimazolam besylate used for endoscopic retrograde cholangiopancreatography. (A) Sequential experimental diagram. (B) Dose–effect curve fitting diagram. [Color figure can be viewed at wileyonlinelibrary.com]

A total of 60 patients were included, 31 females and 29 males, aged between 18 and 65 years. All patients completed their study per protocol (Table 3). During the diagnosis and treatment of ERCP, if cough and body movement occurred during the placement of the duodenoscope, they were determined to be positive reactions, and the dose for the next patient was increased. If no cough and body movement occurred during the placement of the duodenoscope, they were determined to be negative reactions, and the dose for the next patient was decreased. There were 31 positive reactions in all patients and 29 negative reactions. The ED_50_ value of remimazolam besylate was 0.196 mg/kg (95% CI, 0.187–0.206 mg/kg) and the ED_95_ value was 0.239 mg/kg (95% CI, 0.221–0.297 mg/kg) for the procedural sedation of ERCP. The sequential experimental diagram and the dose–effect relationship fitting curve of remimazolam besylate used for ERCP procedural sedation are shown in Figure 2.

**Table 3:** Demographics of the study population (*N* = 60)

Sex (male/female)	Age (years)	Height (cm)	Weight (kg)	BMI (kg/m^2^)
29/31	48.75 ± 11	158.8 ± 6.8	60.08 ± 9.78	23.06 ± 3.41

*Note*: The data are reported as mean ± SD (range).

Abbreviation: BMI, body mass index.

### 3.2 Anesthesia information for the ERCP operation

The average time from the administration of remimazolam besylate to a MOAA/S score ≤ 2 points was 82.58 ± 21.70 s. The average duration of the ERCP operation was 26.25 ± 12.88 min. The duration of anesthesia was 30.42 ± 13.00 min. The average awakening time was 9.03 ± 5.64 min (Table 4).

**Table 4:** Anesthesia information for the ERCP operation

MOAA/S score ≤ 2 points (s)	Duration of operation (min)	Duration of anesthesia (min)	Awakening time (min)
82.58 ± 21.70	26.25 ± 12.88	30.42 ± 13.00	9.03 ± 5.64

*Note*: All data are reported as mean ± SD (range).

Abbreviations: ERCP, endoscopic retrograde cholangiopancreatography; MOAA/S, modified alertness/sedation.

### 3.3 Comparison of the mean arterial pressure (MAP), HR, RR, and SPO_2_ at different time points

Compared with *T*_0_, MAP decreased at T_1_, *T*_2_, *T*_4_, *T*_5_, and *T*_6_ (*p* < 0.05), but the range of decrease was within 20% of the basic value; HR at *T*_1_ and *T*_2_ was higher than that at *T*_0_ (*p* < 0.05), but it did not have clinical significance. HR at *T*_4_, *T*_5_, and *T*_6_ was not significantly different from that at *T*_0_ (*p* > 0.05). RR at *T*_2_ was lower than that at *T*_0_ (*p* < 0.05), but in the normal range; RR at *T*_1_ and *T*_4_ was not significantly different from that at *T*_0_ (*p* > 0.05). SPO_2_ was higher at *T*_4_ than at *T*_0_ (*p* < 0.05), but within the normal range; SPO_2_ at *T*_1_, *T*_2_, *T*_5_, and *T*_6_ was not significantly different from that at *T*_0_ (*p* > 0.05). Due to the limitations of the PACU vital signs monitoring equipment functionality, the respiratory frequency was not recorded at *T*_5_ and *T*_6_ (Table 5). BP in 2 of the 60 patients decreased by more than 30% of the baseline value and all returned to the normal range after the administration of ephedrine. No adverse reactions such as injection pain, respiratory depression, nausea, and vomiting were observed in any of the patients.

**Table 5:** Comparison of MAP, HR, RR, and SPO_2_ at different time points ($\bar{x}\pm \mathrm{SD}$, *N* = 60)

Point of time	MAP (mmHg)	HR (time/min)	RR (times/min)	SPO_2_ (%)
*T*_0_	99.58 ± 13.35	92.10 ± 18.08*	17.63 ± 3.54	97.81 ± 1.38
*T*_1_	87.46 ± 11.74*	100.37 ± 15.92*	16.58 ± 4.56	98.20 ± 1.69
*T*_2_	86.03 ± 13.14*	100.57 ± 14.26	15.58 ± 4.23*	98.30 ± 1.43
*T*_4_	92.02 ± 14.70*	96.58 ± 16.09	18.07 ± 4.54	98.57 ± 1.73*
*T*_5_	90.86 ± 11.99*	94.12 ± 15.70	−	97.37 ± 2.05
*T*_6_	91.14 ± 12.19*	88.77 ± 14.50	−	97.23 ± 1.87

Abbreviations: HR, heart rate; MAP, mean arterial pressure; PACU, postanesthesia care unit; RR, respiratory rate; SPO_2_, pulse oxygen saturation; *T*_0_, basic value after entering the ER; *T*_1_, 2 min after administration of remimazolam besylate; *T*_2_, duodenoscopy; *T*_4_, at the end of diagnosis and treatment; *T*_5_, when waking up; *T*_6_, when leaving the PACU; compared with *T*_0_.

* *p* < 0.05.

## 4 DISCUSSION

ERCP is a common diagnosis and treatment method for biliary and pancreatic diseases.^1^ However, the complexity, duration, and invasive treatment can lead to anxiety, discomfort, and pain in patients. Therefore, deep sedation and general anesthesia are increasingly being used in ERCP diagnosis and treatment, and the success rate of ERCP surgery has improved.^10^, ^11^ At the same time, because ERCP patients are usually in a prone position or a semiprone position, physiological changes in hemodynamics and lung ventilation may occur in this position, which considerably increases the difficulty in respiratory tract management during the operation. ERCP is highly irritating and may cause pain during specific procedures, such as balloon dilatation or stent placement, so sedatives alone do not have sufficient analgesic effects to suppress visceral pain.^12^ Compared with sedatives alone, the combination of opioid analgesics can decrease the pain level, increase practitioner satisfaction, and provide hemodynamic stability.^13^ At present, propofol combined with opioids is commonly used for sedation and anesthesia in clinic, but intravenous propofol induces obvious pain, and exerts certain inhibitory effects on the respiratory and circulatory systems. Propofol infusion syndrome (PIS) increases the risk of cardiovascular complications in patients.^14^, ^15^, ^16^ Remimazolam besylate is an ultra‐short‐acting benzodiazepine, which has the advantages of no injection pain, quick effect, quick recovery, low incidence of hypotension and respiratory depression, etc. It may be a better choice for sedation anesthesia in ERCP surgery, but there are few related clinical data. In this study, we found that the induction of anesthesia with 0.239 mg/kg remimazolam besylate combined with 0.1 μg/kg Sufentanil can almost meet the sedation and anesthesia needs of patients undergoing ERCD surgery. The sequential test is a commonly used test method to calculate ED_50_ and ED_95_ values. For this test, many samples do not need to be obtained, and the test itself is simple and effective. The test results can accurately reflect the potency of drugs. It is one of the classic methods for clinical research of drug dose–effect curves. The qualitative standard of sedation results in this study was the patient's body movements and cough responses during duodenoscopy, so the requirement of gastroscopy for sedation was lower than that of ERCP. In the positive group, three patients were treated with propofol for remedial sedation during ERCP, and all of the symptoms occurred during duodenoscopy. Among them, the first dose of remimazolam besylate in two patients was 0.17 mg/kg. ERCP was successfully performed in one patient after one additional dose of propofol and in the other patient after two additional doses of propofol. In the trial, only 2/10 cases were successfully treated with 0.17 mg/kg during duodenoscopy, with a success rate of 20%; this indicates that this initial dose may not meet the requirements for ERCP sedation. In another patient, the first dose of Remimazolam besylate was 0.21 mg/kg, and the diagnosis and treatment were successfully carried out after propofol was administered once for remedial sedation. In the study, although MAP was lower than the basic value at T_1_, T_2_, T_4_, T_5_, and T_6_ time point, the decrease range was less than 20% of the basic value. The HR was higher than the basic value at 2 min after administration and at the time of duodenoscope insertion, but it had no clinical significance. RR and SPO_2_ were in the normal range, so the anesthetic dosages used in ERCP treatment had a slight impact on patients' breathing and circulation. In two patients, the BP decreased by more than 30% of the basic value after the administration of ephedrine, and the BP increased to the normal range after the administration of ephedrine, which may be related to the long duration of fasting and the relatively insufficient capacity.

A Phase III clinical study showed^17^ that both remimazolam and propofol can lead to the expected sedative effect in upper gastrointestinal endoscopy. The incidences of pain, hypotension, and respiratory depression caused by remimazolam injection are lower than that caused by the use of propofol. A clinical study showed^18^ that lost of consciousness was observed (5 ± 1) min after start, and full alertness was regained (19 ± 7) min after stop of infusion, and the clearance rate of remimazolam was (1.15 ± 0.12) L/min. Therefore, in this study, remimazolam besylate was used in the diagnosis and treatment of ERCP to achieve the ideal sedative effect and reduce the incidence of adverse reactions such as hypotension and respiratory depression. In clinical practice, ERCP diagnosis and treatment involve the use of a duodenoscope, and the use of a traditional gastroscope provides a direct view, because a gastroscope is thinner than a duodenoscope, thus making ERCP diagnosis and treatment more difficult than that with the use of a gastroscope. Using remimazolam besylate for anesthesia induction not only meets the needs of sedation anesthesia in surgery but also considerably reduces the incidence of related adverse events, which is a selective scheme of anesthesia in ERCP surgery.

In conclusion, the ED_50_ and ED_95_ values of remimazolam besylate used in the procedural sedation of ERCP were 0.196 and 0.239 mg/kg, respectively, with definite sedative effects and little influence on respiration and circulation, so this study provides a reference for safe medication in clinical practice.

## AUTHOR CONTRIBUTIONS

Dan‐Dan Tan contributed to the experimental design, research, and writing of the article. Jin Gu, Li‐Jin Zhao, and Guo‐Hua Zhu contributed to the research. Juan Li was responsible for experimental guidance and quality control. De‐Xing Liu, Xin‐Xin Yang, and Jin Tian contributed to data analysis and the creation of figures. Wan‐Qiu Yu contributed to participants' follow‐up in PACU and wards. Qi Tian provided some help with the revision of the article. Zhao‐Qiong Zhu was responsible for experimental guidance and problem‐solving.

## CONFLICT OF INTEREST

Jin Tian is a member of *Ibrain*'s editorial board, but is not involved in the peer‐review process of this article. The remaining authors declare no conflict of interest.

## TRANSPARENCY STATEMENT

All the authors affirm that this manuscript is an honest, accurate, and transparent account of the study being reported; that no important aspects of the study have been omitted; and that any discrepancies from the study as planned (and, if relevant, registered) have been explained.

## ETHICS STATEMENT

All the experiments on the included participants were approved by the Ethics Committee of Biomedical Research for Affiliated Hospital of Zunyi Medical University (no. KLL‐2020‐280). This trial had been registered at chictr.org.cn (no. ChiCTR2200059783) and was conducted following the Declaration of Helsinki.

## Data Availability

The authors confirm that the data supporting the findings of this study are available within the article and its Supporting Information: Materials.
